# Fecal Cortisol Metabolites in Dairy Cows: A Cross-Sectional Exploration of Associations with Animal, Stockperson, and Farm Characteristics

**DOI:** 10.3390/ani10101787

**Published:** 2020-10-01

**Authors:** Asja Ebinghaus, Ute Knierim, Christel Simantke, Rupert Palme, Silvia Ivemeyer

**Affiliations:** 1 Farm Animal Behaviour and Husbandry Section, University of Kassel, Nordbahnhofstr. 1a, 37213 Witzenhausen, Germany; uknierim@uni-kassel.de (U.K.); simantke@uni-kassel.de (C.S.); ivemeyer@uni-kassel.de (S.I.); 2 Unit of Physiology, Pathology and Experimental Endocrinology, Department of Biomedical Sciences, University of Veterinary Medicine, Veterinärplatz 1, 1210 Vienna, Austria; rupert.palme@vetmeduni.ac.at

**Keywords:** dairy cows, stress, cortisol metabolites, human–animal relationship

## Abstract

**Simple Summary:**

Dairy cows are exposed to various potentially stressful situations in the daily farm routine, which might considerably impair their welfare and performance. On 25 German organic dairy farms, we explored associations of cows’ physiological stress levels by means of cortisol metabolite concentrations in feces (1) with different potentially influencing farm factors including human–animal contact, (2) cows’ fear behaviors towards humans, and (3) milk production and udder health. Cortisol metabolite levels were decreased on farms that did not separate diseased cows, possibly reflecting less regrouping stress. Levels were also lower on farms with straw yards compared to raised cubicles, and on farms with generous compared to suboptimal lying space, underlining the importance of resting comfort for cattle. Increased human–animal contact was associated with decreased cortisol metabolite levels. However, against expectations, levels were higher, when the farm provided concentrate feed by hand and habituated young cows to milking, requiring specific experimental investigations to draw conclusions on causal associations.

**Abstract:**

To date, little is known about influences on cows’ physiological stress levels on farms. The present study explored associations of fecal cortisol metabolite concentrations (FCM) with (1) farm factors including human–animal contact, (2) cows’ fear behaviors towards humans, and (3) milk production and udder health, involving 25 dairy farms and repeated fecal samples (*n* = 2625) from 674 focal cows. Farm factors via interviews and observations, avoidance distance (AD) and qualitative behavior assessment (QBA) during a human–animal interaction were recorded. Milk yield and somatic cell scores (SCS) were calculated from milk recordings. Levels of FCMs were in general relatively low. No associations with AD and milk yield could be detected. Correlations between FCMs and QBA and SCS were significant, but on a low level. Against expectations, FCMs were higher, when the farm provided concentrates by hand and habituated heifers to milking, in part possibly due to reversed cause–effect relations. Decreased FCM levels were found on farms that did not separate diseased cows, possibly due to the avoidance of social stress following changes in group structure. Additionally, straw yards compared to raised cubicles and generous compared to suboptimal lying space were associated with decreased levels, underlining the importance of comfort around resting. Moreover, FCMs were decreased with increased human contact time per cow. The different associations detected in this study provide a basis for further experimental investigations that moreover might provide insights into causal relationships.

## 1. Introduction

In dairy farm systems cows are exposed to high social and physiological demands, including different potentially stressful situations in the daily routine, such as changing group structures, separation from conspecifics, frequent handling by humans, e.g., during milking, or potentially aversive routine procedures involving restraint, novelty, or noise, e.g., during veterinary procedures [1] or claw trimming [2,3].

When an animal experiences a stressful situation, a number of behavioral and physiological responses can be activated to help the organism to cope with temporal stressors [4]. Sustained stress, however, has been found to be related to reduced productivity and increased disease susceptibility in cows: investigations by Holtenius et al. [5], for instance, indicated associations between metabolic stress indicators and incidences of mastitis treatments. More recently, Ivemeyer et al. [6] found lower physiological stress responses associated with increased mastitis curing rates during lactation.

One central component in the animal’s physiological stress response is the secretion of glucocorticoids (particularly cortisol in cattle) from the hypothalamic–pituitary–adrenal (HPA) axis with a major role in energy regulation. Thus, concentrations of glucocorticoids (GCs) or their metabolites are used as stress indicators and have accordingly been extensively used in animal welfare research (reviewed in [7,8]). Besides the traditional method of measuring GC concentrations in blood serum/plasma, also noninvasive matrices are used, e.g., saliva, milk, or urine [9]. A well-established noninvasive method to assess stress levels over longer periods is to analyze fecal cortisol metabolites (FCMs) by an enzyme immunoassay (EIA), developed by Palme and Möstl [10]. In contrast to cortisol measures in blood or saliva, FCMs reflect the cortisol secretion over a longer period, with a delay of the gastrointestinal passage rate of 9–15 h in cattle, and thus, are less prone to short-term variations during the day (reviewed in [7]).

Applied properly, FCMs are considered a powerful tool. Although a number of methodical challenges need to be observed [7], previous investigations showed that different short-term and prolonged or recurrent stressors can be related to an increase of FCM concentrations: Palme et al. [11], for instance, demonstrated that a 2 h road transport of cows was followed by a significant increase in FCM concentrations. Möstl et al. [12] described that after transport into an unfamiliar stable the cortisol excretion via feces of test cows was elevated for about 1 week and declined thereafter to baseline values. In further investigations, FCM measures have been used to detect mid-term stress after claw trimming [3] and during a 14-day-period of overstocking [13], as well as chronic or recurrent stress when providing cows inappropriate lying surfaces [14]. Moreover, FCMs have been found to be associated with the prevalence of hock-lesions [15], which again could be attributed to chronic or recurrent stress due to suboptimal housing design.

In addition to the aforementioned potential stressors, also human–animal contacts during routine work can be a major source of stress in farm animal species. Previous investigations on the human–animal relationship showed associations between stockpersons’ attitudes and behaviors towards the animals and the animals’ levels of fear of humans, measured e.g., by means of cows’ avoidance distances towards an experimenter [16,17]. Indications for associations of the increased fear levels with physiological stress responses are their effects on milk ejection and milk yield [18,19,20], success at first insemination [21], and aspects of udder health [6,22]. Cows’ acute physiological stress responses in the presence of humans have been investigated by Hemsworth et al. [23], who found lower milk cortisol concentrations in primiparous cows that had been handled positively during calving compared to those without additional human handling. Additionally, Breuer et al. [24] reported effects of positive and negative handling on dairy heifers’ blood cortisol concentrations in the presence of humans. More recently, Lürzel et al. [25] found significantly lower salivary cortisol values before and after an isolation test in calves, which had experienced 40 min of gentle interactions (stroking and gentle talking) during the first 4 weeks of life, compared to control calves. A number of studies in pigs indicated chronic stress responses in animals that are fearful towards humans (e.g., [26,27]). In cattle, however, mid- or long-term effects of the quality or quantity of human–animal contact have not yet been investigated.

**Figure 1:**
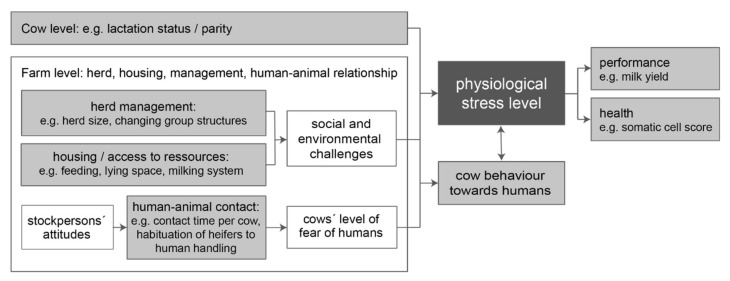
Potential associations between animal and farm factors, cows’ physiological stress levels, milk performance, and udder health (factors with gray background were considered in the present investigation).

Furthermore, to date no cross-sectional investigations have been conducted to explore the interplay of different potentially influencing factors (Figure 1) on the cows’ physiological stress levels, including characteristics of herd, housing, management, and human–animal contact.

The present cross-sectional investigation aimed at exploring the mid-term physiological stress level by means of FCMs in lactating cows on different German organic dairy farms. In detail, we aimed to explore possible (1) important influences of management, housing, and human–animal contact on FCM levels, while taking potentially confounding effects of cow lactation status (DIM) and the day time of sampling into account, (2) associations between cows’ fear behaviors towards humans and FCM levels, and (3) associations between FCM levels and milk yield as well as udder health (somatic cell scores, SCS).

## 2. Materials and Methods

### 2.1. Farms and Animals

The investigation was implemented within the European CORE Organic Plus project OrganicDairyHealth and conducted in close collaboration with a German national project within the interdisciplinary LOEWE research cluster ‘Animals – Humans – Society’. For the present examination, data of 25 organic dairy farms located in Middle and Northern Germany were included.

The investigation was carried out in compliance with the German animal welfare act; it did not involve aversive animal handling. Collection of fecal samples and observations of animal behavior were conducted under unchanged farm conditions. Fecal samples were mainly collected after defecation without animal handling (84%), the others were taken rectally with due care. The participation of farms in the study was voluntary, and the farmers were informed about the purpose and methods of the study by written project information in advance. They were assured that all information would be treated anonymously, and that they could withdraw from the study at any time. For the use of herd data, written farmers’ consents were obtained.

Farm visits for data collection were conducted during the winter periods of 2015 and 2015/2016. Assessments were done during winter to enable observations of cow behavior in the barn and to exclude direct effects of pasture access.

Although the farm sample was a convenient sample, some criteria were considered in the selection process: all farms kept mainly (>50%) or exclusively Holstein Friesian or Red Holstein cows in loose housing systems (cubicles or straw yards) and participated in official milk recording schemes. Apart from these criteria, farms were selected to cover a typical range of different dairy farm conditions, particularly regarding herd size and milking system. Herd sizes ranged from 29 to 161 cows (mean ± sd = 69.4 ± 30.8); the average daily milk yield ranged from 13.6 to 25.9 kg (mean ± sd = 21.7 ± 2.8). Five farms used automatic milking systems (AMS), the others milked in fishbone (16 farms) or tandem parlors (four farms). Summer pasture was offered for all cows on 23 farms, on one farm only for dry cows, and one farm had zero grazing. The majority of farms (16 farms) were family operated, while the others were farm communities. Horned cows were kept by 12 farms, the others kept dehorned or partly genetically hornless cows. Further details on farm characteristics are given in Table 1.

### 2.2. Data Collection

#### 2.2.1. Herd, Housing, Management, and Human–Animal Contact

Herd and housing factors as well as management characteristics (including herd and milking management, feeding regime, and human–animal contacts) of the farms were assessed via questionnaire-guided interviews (by one of in total two researchers) and observations during the farm visits. Regarding human–animal contact during routine work the number of cows per stockperson, contact time “on foot” per cow (including milking), habituation of heifers to humans, and frequency of control rounds in the barn were recorded. Moreover, voluntary human–animal contacts with lactating cows and dry cows beyond routine work were recorded. These voluntary contacts were quantified according to the weekly frequencies of different interactions (observing, brushing, speaking to animals, and udder control beyond milking). Each interaction was multiplied by a factor, depending on the frequency stated by the stockpersons (categories: daily, every 2–3 days, once a week, rarer, never)—ranging from factor 4, if carried out daily, to factor 0, if never carried out. Thus, each farm could reach up to 16 points. The measure was expressed as the percentage of actual points in relation to the maximal points. Moreover, during the farm visits stockpersons ability to identify individual cows was observed.

#### 2.2.2. Cows’ Physiological Stress Levels (FCMs)

For the assessment of the physiological stress level by means of fecal cortisol metabolites (FCMs), 23–36 focal cows per farm (mean ± sd = 27.9 ± 5.9), depending on herd size, were selected. The following selection criteria were applied: as equal distributions of primiparous cows, cows in the second or third lactation, and older cows per farm as possible, and focal cows should not be more than 200 days in milk (DIM) at the first sampling date. Up to four repeated fecal samples per cow were collected (serial sampling) with a time interval of 5–15 days (mean ± sd = 8.3 ± 2.2). Cows with overt clinical symptoms were not selected as focal animals. However, if an animal showed any observable clinical symptoms during sampling, this was noted additionally.

In total, 2635 fresh fecal samples of 698 focal cows were collected directly after defecation or sometimes rectally, filled into small tubes, and stored in a mobile freezer (−18 °C) immediately or 1 h after collection at the latest. After return from the farm visit, the tubes were stored at maximum 6 months in a deep freezer (−25 °C) until transport to the laboratory of the University of Veterinary Medicine, Vienna. Samples were then extracted by using the wet extraction method (0.5 g feces plus 5 mL 80% methanol) described by Palme (2005) and Palme et al. (2013). FCMs were analyzed with an 11-oxoetiocholanolone EIA, developed and described in detail by Palme and Möstl [10]. It measures 11,17-dioxoandrostanes (11,17-DOAs), a group of cortisol metabolites, and has been validated for the assessment of adrenocortical activity in cattle [8,28]. High and low concentration pool samples were run within each assay, and resulting intra- and interassay coefficients of variation were always below 10% and 15%, respectively.

For later statistical analyses, FCM values ≤ 2.2 ng/g (10.7% of all values) were set to the detection limit of 2.2 ng/g. Furthermore, only cows with at least three of four repeated measurements (674 of 698) were integrated to avoid bias caused by individual cows, which had left the herd during the project period e.g., for health reasons. Five of 674 focal cows were lame at one of the sampling dates (none of the focal cows were lame at more than one date), and 18 of 2635 samples were thin feces. Since these samples did not show any major deviations with regard to FCM concentrations, the values were not excluded. After data cleaning, sample sizes per herd varied between 16 and 36 (mean ± sd = 27.0 ± 5.6). In total, 584 of 674 focal animals (86.6%) were Holstein cows, thereof 401 Holstein Friesian, 152 Red Holsteins, 28 German Red Pied, and three German Black Pied. The others were of the breeds Jersey, Brown Cattle, Red Angler, Fleckvieh, or crossbreeds.

For part of the univariable analyses on animal level, the median of the three or four repeated FCM measurements was calculated; for analyses at farm level, the herd medians were calculated from the animal medians.

#### 2.2.3. Cows’ Behaviors towards Humans

The cows’ fear behaviors towards humans were assessed at individual animal level by direct observations once during the first farm visit, when also the first fecal samples were taken. Avoidance distances (AD) were measured and QBA was conducted during a standardized handling situation as described by Ebinghaus et al. [29]. Behavioral observations were conducted by seven trained observers altogether. Prior to data collection, interobserver reliabilities for all observers had been tested and acceptable agreements achieved (Spearman rank correlations = 0.71–0.94).

Assessments always started after feeding in the morning, when the majority of cows were restraint in self-locking feeding gates. The tests were applied at the feeding place, always sequentially in the same order on each focal cow. At first, the AD was recorded: one experimenter located on the feed bunk outside the barn approached the restrained test cow from 2 m distance at a speed of one step per second with the arm held overhand in an angle of 45° from the body, and recorded the distance at which the animal withdrew (in 10 cm increments) or if the muzzle could be touched (0 cm). Subsequently, another experimenter, located on the barn side of the feeding gate and blind to the assessment of AD, performed a standardized human–animal interaction: the experimenter looked at the fixed test cow from behind, left and right hand side for about 30 s, then approached the animal from one side, stroked three times along the back and down the flank, and opened the feeding gate to release the cow from restraint. Afterwards, the experimenter scored the cow’s expressive behavior during the entire interaction using visual analogue scales on a given list of 20 descriptors, which had been developed for this purpose [29] based on Wemelsfelder et al. [30]. The multivariate QBA data of all individually assessed cows were reduced to two dimensions by means of principal component analysis (PCA, eigenvalue > 1, correlation matrix, without rotation, SPSS 24). The PCA explained 67.5% of variance on the first principal component (PC1), which was characterized by descriptors relating to relaxation/attraction/trust on the negative and descriptors relating to fear/distress/aversion on the positive end. For further analysis, the cows’ individual scores of the PC1 were used.

#### 2.2.4. Cows’ Milk Yield and Udder Health

The individual cow’s lactation number (LN) and days in milk (DIM) at the first sampling date were taken from the milk recording data (MRD). Based on the monthly MRD test day results recorded during the time period from 14 d before first sampling date to 14 d after last sampling date, the average daily energy corrected milk yield (ECM), and somatic cell score (SCS) were calculated.

### 2.3. Statistical Analyses

To identify important influencing factors on the outcome variable of FCMs (objective 1) according to Figure 1, a linear mixed-effects model was performed at sample level using the lmer-method from the lme4 package in R studio, version 1.2.1335 for Mac OS X. For model fitting, a minimum model including the fixed effects of day time of sampling and DIM and the random effect of the individual animal nested in the farm was set up. The fixed factors listed in Table 1 were tested in a forward procedure, and included in the model depending on the Akaike information criterion (AIC).

Model diagnostics (normal distribution, homoscedasticity) were done by graphical evaluation of the residual distribution and the residuals-by-predicted-values plot. To satisfy model assumptions, FCM values needed to be transformed by x^0.2. For each factor included, the estimate and 95% confidence intervals, *p*-value, and effect size are reported. Effect sizes were calculated with r = √ (t^2^/(t^2^ + df)) (t = value of the t-statistic used to calculate *p*-values, df = degree of freedom). Values < 0.1 were referred to as very low, values ≥ 0.1 < 0.3 as low, values ≥ 0.3 < 0.5 as moderate, and values ≥ 0.5 as strong [31]. Absence of multicollinearity was checked using the variance inflation factor (VIF, < 4.0); factors correlating strongly with each other (r_s_ > 0.70, Appendix Table A1) were not included together in the model. The absence of influential data points was checked using Cooks’ distance (<0.1).

Univariable associations between cows’ behaviors and FCMs (objective 2), and cows’ FCMs and milk yield as well as udder health (objective 3) were analyzed using nonparametric Spearman rank correlation (r_s_), because graphical tests via normal quantile-quantile plots [32] showed that data (e.g., AD, QBA) were partly non-normally distributed.

## 3. Results

### 3.1. Descriptive Data

Descriptive data of animal-, herd-, and farm-related factors potentially associated with FCM levels are shown in Table 1. The investigated focal cows varied between the first and twelfth lactation. On average they were in milk for 92.3 ± 51.5 days. All factors were considered in the multivariable analysis.

Concentrations of FCMs varied markedly, both between individual animals and between herds. Descriptive results regarding data of the first sampling and of averages of all 3–4 sampling dates are presented in Table 2.

### 3.2. Influences on FCM Levels

In addition to the given covariables (day time of sampling and DIM), six further farm factors relating to herd management, housing, and human–animal contact were integrated in the final multivariable model. Effect sizes of the covariables were very low for the day time (0.03–0.09) to low for the number of DIM (0.14); effect sizes of the farm factors ranged from moderate to strong (Table 3).

### 3.3. Associations of FCM Levels with Cows’ Behaviors towards Humans, Cows’ Milk Yield and Udder Health

No associations of FCM levels with AD or milk yield was detected. Correlations with QBA and SCS were significant, but on a low level (Table 4). Table 4 further shows descriptive data of cows’ behaviors towards humans, milk yield, and udder health at animal level.

## 4. Discussion

The present investigation aimed at examining associations between fecal cortisol metabolites (FCMs) reflecting the cows’ mid-term physiological stress levels and farm factors, the cows’ behaviors and milk yield as well as udder health. Particular attention was paid to factors of human–animal contact.

### 4.1. Level and Range of FCM Levels in the Investigated Sample

Since measurements of GC metabolites in fecal samples strongly depend on the methods used, but analytical methods vary between different laboratories, opportunities to compare between investigations are limited. Higher concentration levels found do not necessarily indicate higher adrenocortical activities (i.e., a stronger stress response), but may have their basis in methodical issues [7]. For example, time periods and temperatures at sample collection and storage have to be considered [33]. Thus, meaningful comparisons, also within the same species, are only possible, if exactly the same methods were used [8].

The medians of repeated FCM measures found in the present investigation ranged on a relatively low level between 2.2 and 47.6 ng/g at animal level (median = 11.0 ng/g, mean = 12.2 ng/g) compared to previous investigations using the same method (e.g., [15,34,35]): Rouha-Mülleder et al. [15] investigated singularly measured FCM levels on 80 dairy farms and found concentrations at farm level ranging from 30 to 157 nmol/kg (median = 77 nmol/kg, corresponding to approx. 23.4 ng/g). In an investigation exploring dairy cow coping capacity during a change from conventional to automatic milking, Weiss et al. [35] measured average FCM concentrations ± sd of 134 ± 12 ng/g at animal level during the control period. Before dry-off, Bertulat et al. [34] measured baseline FCM concentrations in 80 late lactating dairy cows ranging from 30.0 to 184.9 ng/g at animal level. The maximum values correspond to some higher values that we found at sample level (max = 159.5 ng/g). Lower FCM values in nonlactating dairy cows in New Zealand, ranging from 6.0 to 8.2 ng/g [14], resulted from a commercial corticosterone assay, thus values cannot be directly compared.

Due to considerable differences between extraction and assay methods, no threshold or target has been defined for FCM measures in cattle, so far [7]. However, it appears that the results of the present investigation range on a relatively low level.

### 4.2. Identified Factors Influencing FCM Measures

The multivariable modelling resulted in a final model including altogether eight factors. As potential confounders, day time of sampling and cow’s DIM were integrated as fixed factors in the minimal model and remained significant (*p* < 0.001) in the final model. However, effect sizes of day times were very low (ranging between 0.03 and 0.09) and low for DIM (0.14), suggesting that these factors should be considered, but did not strongly affect the present analyses. This may be explained by only a weak increasing effect of advanced pregnancy [7] in the sample, since late lactating cows (>200 DIM at the first sampling date) were not included, and part of the cows being in early lactation and not yet pregnant again. Physiological changes and challenges in early lactation may have even counteracted: Fukasawa et al. [36] found higher cortisol concentrations measured in milk samples of cows in early lactation (7–90 DIM: 0.39 ng/mL) compared to cows in later lactation stages (e.g., 91–180 DIM: 0.22 ng/mL).

The factors included by means of forward selection, referred to different aspects of management, housing, and human–animal contact. Three factors relating to human–animal contact were included in the final model: contact time per cow, active habituation of heifers to milking, and manual provision of concentrates. The contact time per cow describes the quantity of human–animal contact during routine work in min/d. Higher contact times were associated with lower FCM levels, suggesting that prolonged contact to humans can decrease cows’ overall stress levels. One reason for this could be reduced fear towards humans due to an increased quantity of human–animal contact of positive quality as found in earlier investigations [16,37,38]. However, in the present study no substantial association between FCM levels and fear-indicating responses in the avoidance distance (AD) test and regarding the cows’ expressive behaviors during a standardized human–animal interaction (QBA) could be detected. Therefore, reduced cows’ fear responses towards humans appeared to be less crucial here. Further mechanisms, such as earlier detection and solving of e.g., technical or social problems in the herd due to more human presence in the stable, need be considered, too.

Although provision of attractive feed by hand is considered a pleasant human–animal interaction and was shown to be associated with less fearful responses towards humans [39], manual concentrate provision was associated with increased FCM levels in the present investigation. This might be explained by increased arousal, physical activity [40], and possibly also social stress during feeding, when the attractive feed is provided.

Active habituation of heifers to the milking parlor or automatic milking system (AMS) reported by the farm managers was associated with increased FCM levels. Similarly, in a previous investigation of the present data on farm level, Ivemeyer et al. [6] found active habituation of heifers to milking associated with impaired udder health. Possibly, extra habituation efforts reflect a necessity due to problems with nervous heifers rather than additional positive human–animal contact.

From the field of investigated management factors, increased FCM levels were found in herds where diseased or lame cows were separated from the herd. Although for the diseased or lame cows the separation might provide some protection and promote recovery, apparently a disadvantage can be the associated changes of group structure which may increase social stress: Von Keyserlingk et al. [41] observed that regrouping of dairy cows can disrupt behavior and impair production over days following the event, suggesting an impact of stress due to increased agonistic interactions.

With regard to housing, two factors were included in the final model: housing type and cow:lying space ratio. FCM levels were significantly lower on farms with straw yards compared to farms with raised cubicles. Additionally, Palme et al. [42] and Fisher et al. [14] found differences in FCM levels of cows housed in different systems. Fisher et al. [14] analyzed FCMs from nonlactating cows that had been moved from pasture to four different floor types for four days, respectively. The lowest concentrations were measured when the animals were kept on a deformable floor; the highest concentrations were found when they were kept on concrete floor or on a section of the farm laneway. Accordingly, Palme et al. [42] showed that cows on straw yards had significantly lower FCM values than those housed in systems with mainly raised cubicles.

In the present sample, associations found regarding cows’ access to resources raise several questions: the significantly lower FCM levels on farms offering a generous cow:lying place ratio compared to farms offering a suboptimal ratio conformed to expectations. However, FCM levels were higher when the animals had access to currently recommended lying place provision compared to suboptimal space. The same pattern was visible in the data regarding the cow:feeding place ratio. This result may call the current recommendations into question where partly even less than one lying or feeding place per cow are foreseen. However, the lower FCM levels under suboptimal conditions might be explained by nonlinear associations: typically, GC levels increase in response to a challenge or stressor, and thus, as a rule high levels can indicate stress. However, low levels can also be attributed to a downregulating response after long-term exposure to stressors. For instance, a recent investigation found lower plasma cortisol and FCM concentrations in domestic horses showing poor welfare in terms of depressive-like behaviors compared to the control animals [43]. Inconsistent patterns and nonlinear associations were also found in wild animal species with regard to associations with reproduction rate and fitness [44].

### 4.3. Associations between FCMs and Cow Behavior

Although the cows’ fear behaviors towards humans ranged markedly on the investigated farms, both measured by AD (0.0–170.0 cm) and QBA (−1.713–2.355), no association between the cows’ FCM levels and AD was found. Additionally, the correlation to QBA was almost negligible, though significant (r_s_ = −0.149, *p* = 0.008). The direction of this correlation is contrary to expectations, but should not be over-rated because of the low effect size.

Previous investigations that found associations between positive handling treatments and the animals’ physiological responses in the presence of humans used GC measures reflecting the acute stress level, i.e., cortisol concentrations in milk [23], blood [24], and in saliva [25]. Apparently, in animals that are more fearful towards humans, human–animal contacts can result in an immediate release of stress hormones. However, the present results suggest that this is not reflected in the general physiological stress level, with FCM levels reflecting the cortisol secretion over a longer period [4].

### 4.4. Associations between FCMs and Milk Yield and Udder Health

Associations between FCM level and milk recording data were investigated to quantify potential effects on cows’ health and milk production. Only a slight, but significant positive correlation between FCM concentrations and somatic cell scores at test days as close as possible to the feces sampling was found in the present investigation (r_s_ = 0.109, *p* = 0.005). A previous multivariable analysis of the present data on farm level regarding effects on udder health showed that lower median herd FCM levels were related to enhanced mastitis cure rates [6]. The current results reflect only a low general influence of stress on udder infections that relate to immunosuppressive effects of longer lasting increased GC levels.

The daily milk yield (energy corrected milk, ECM), which was on average on a very moderate level in the investigated sample, was not related to the cows’ FCM levels. Previous investigations showed heterogeneous results: Rouha-Mülleder et al. [15] found FCM levels negatively correlated with the daily milk yield. According to their interpretation, distress might have led to increased amounts of residual milk. Additionally, in a study of Bertulat et al. [34] regarding stress after sudden dry-off, high-yielding cows had the lowest baseline value of FCM concentrations before drying-off and low yielding cows had the highest concentrations. In contrast, Pesenhofer et al. [3] and Fukasawa et al. [36] found no significant correlation between milk yield and FCM basal values. Major influences on milk yield are the animal’s genetic potential [45,46], the feeding level [47,48,49,50], lactation stage [51], and health [52,53] whose joint effects on GC levels are not clear-cut. Therefore, within herd or well controlled investigations of possible associations might produce more meaningful results regarding possible stress effects on performance than cross-sectional designs.

### 4.5. Limitations of the Study

Using FCM measures in order to evaluate the physiological stress level is a noninvasive alternative that has been validated and repeatedly applied in cattle (reviewed in [8]). However, concentrations of FCMs measured by means of immunoassays always have to be interpreted as relative values. Furthermore, not every elevated value of FCMs or other measures of glucocorticoid concentrations can be interpreted as distress [54], since also events such as mating (in cattle: [55,56]; in horses: [57]) or environmental enrichment (in pigs: [58]) can increase cortisol levels. Consequently, it is not possible to define absolute threshold values. Furthermore, uncertainties still exist regarding the interpretation of low FCM values, as also found in the present investigation. U-shaped associations due to downregulating processes as a consequence of prolonged distress [44] cannot be ruled out.

Beside the measure used, also the cross-sectional approach implies limitations: on-farm investigations always involve a great number of varying factors and factor combinations so that the associations found, especially in relatively small samples like in the current study, should not be generalized without further external evidence. Moreover, the broad number of potential influencing factors leads to methodical uncertainties regarding their selection within the multivariable modelling. Including or excluding a single factor into or from statistical modelling can result in a different combination of factors included in the final model. In addition, associations with FCM values may be interlinked with a combination of further potential influencing factors that have not been considered, but affect results in an opposite direction.

Thus, cross-sectional studies such as the present investigation can only identify patterns of associations. To prove causal relationships requires specific experimental investigations under reasonably controlled conditions.

## 5. Conclusions

The present cross-sectional investigation showed that FCM measures reflecting the physiological stress level in dairy cows are not straightforward to interpret. With, in general, relatively low FCM levels, no substantial associations on individual level with fear-indicating behavior towards humans, milk yield, or udder health could be detected. The multivariable analysis of external influences on the cows’ FCM levels on farm level yielded some unexpected results which show that the multifaceted nature of stress physiology is difficult to grasp under on-farm conditions. For instance, farms that separated diseased or lame cows had higher FCM levels, possibly because the associated group changes are disadvantageous. Additionally, farms that fed concentrates by hand and that actively habituated heifers to the milking procedure had higher FCM levels, in the latter case possibly because they had more nervous heifers that needed habituation. In addition, a suboptimal animal to lying place ratio was associated with lower FCM levels than lying place provision according to current recommendations. Here, a downregulating response after long-term exposure to stressors might have played a role. However, conforming to expectations, results indicated that generous resource provision and higher comfort around resting as well as increased human contact time per cow contribute to lower physiological stress levels.

## Figures and Tables

**Table 1:** Descriptive data on herd, housing, and management characteristics at farm level (*n* = 25), and on cows’ parity and days in milk (DIM) at animal level (*n* = 674).

Variables	Recording ^1^	Descriptive Data	
Herd Characteristics		Mean ± sd	Median (Min–Max)
Parity (n)	MRD	3.2 ± 2.1	3.0 (1.0–12.0)
DIM (d)	MRD	92.3 ± 51.5	89.5 (1.0–200.0)
Herd size (n)	MRD	69.4 ± 30.8	68 (29–161)
Max. number of cows per group (n)	o	56.2 ± 20.8	57 (29–95)
		**Groups**	**Number (%)**
Cow groups (n)	o	1 (all cows in one group)	5 (20)
		2 (dry cows separated)	13 (52)
		>2 groups	7 (28)
**Housing Characteristics**		**Groups**	**Number (%)**
Housing type	o	Raised cubicles	8 (32)
		Deep bedded cubicles	8 (32)
		Straw yards or mixed ^2^	9 (36)
Cow:cubicle ratio or lying space ^3^ (m^2^/cow)	o	Suboptimal	10 (40)
		According to recommendations	11 (44)
		Generous	4 (16)
Cow:feeding place ratio ^4^	o	Suboptimal	7 (28)
		According to recommendations	9 (36)
		Generous	9 (36)
**Management Characteristics**		**Mean ± sd**	**Median** **(min**–**max)**
Concentrates (kg/cow*year)	i	1203 ± 521	1200 (0000–2000)
		**Groups**	**Number (%)**
Routine fixation for feeding	i	No	11 (44)
		Yes	14 (56)
Concentrates station (in the barn)	o	No	13 (52)
		Yes	12 (48)
Separation of dry cows	i	No	6 (24)
		Yes	19 (76)
Separation of cows in heat	i	No (or rarely)	16 (64)
		Yes	9 (36)
Separation of diseased or lame cows	i	No	7 (28)
		Sometimes	11 (44)
		Yes	7 (28)
Milking system	o	AMS	5 (20)
		Fishbone parlor	16 (64)
		Tandem parlor	4 (16)
Selection of cows for docility	i	No	18 (72)
		Yes	7 (28)
**Human–Animal Contact**		**Mean ± sd**	**Median** **(min**–**max)**
Number of cows per stockperson ^5^ (n)	i	19.4 ± 10.0	18 (4.4–40.5)
Contact time per cow ‘on foot’ ^6^ (min/d)	i	9.0 ± 7.6	6.0 (1.7–32.6)
Voluntary contact to cows (%) ^7^	i	48.3 ± 13.1	50.0 (12.5–68.8)
Voluntary contact to dry cows (%) ^7^	i	50.0 ± 18.7	50.0 (18.75–81.3)
		**Groups**	**Number (%)**
Active habituation of heifers to human handling	i	No	15 (60)
		Yes	10 (40)
Active habituation of heifers to the milking parlor / AMS	i	No	14 (56)
		Yes	11 (44)
Frequency of control rounds in the barn	i	At maximum once a day	7 (28)
		Several times a day	18 (72)
Roughage provision	i	Only by machine	9 (36)
		(Also) manually	16 (64)
Concentrates provision (in the barn)	i	Only by machine/station	15 (60)
		(Also) manually	10 (40)
Stockpersons know individual cows	o	Not all cows	15 (60)
		Yes	10 (40)

^1^ type of data recording: MRD = taken from milk recording data; o = assessed via observation during the farm visits; i = assessed via questionnaire-guided interviews. ^2^ six farms with straw yards and three farms with a combination of straw yards and deep bedded cubicles. ^3^ based on recommendations cited in the literature (details in Appendix Table A2 and Table A3). ^4^ based on recommendations cited in the literature (details in Appendix Table A4 and Table A5). ^5^ incl. herd managers, employees, trainees. ^6^ including milking, excluding time near the cows, but on machines. ^7^ based on weekly frequencies of different human–animal interactions beyond routine work listed in the questionnaire and named by the interviewed farmers (observing animals, speaking to animals, brushing, and udder control).

**Table 2:** Fecal cortisol metabolite (FCM) concentrations (ng/g) at individual animal level (FCManimal) and at herd level (FCMherd), a. values of the first sampling (when cows’ behaviors were also recorded), b. medians of all (3–4) samplings.

FCManimal	n	Mean ± sd	Median	Min–Max	25–75% Percentile
a.	671	13.95 ± 13.45	10.36	2.20–159.50	4.95–19.69
b.	674	12.20 ± 6.90	11.03	2.20–47.60	6.86–15.82
**FCMherd**	**n**	**Mean ± sd**	**Median**	**Min–Max**	**25–75% Percentile**
a.	25	11.42 ± 6.13	12.53	2.20–26.21	6.35–15.33
b.	25	11.15 ± 3.90	10.79	4.42–17.53	7.91–15.45

**Table 3:** Final linear mixed model regarding FCMs (ng/g; data transformed by x^0.2); fit by maximum likelihood, random effect: animal nested in farm; number of samples: 2625; number of groups: animals = 674, farms = 25.

Fixed Effects	Estimate	CI 2.5%	CI 97.5%	*p*-Value	Effect Size
Day time of sampling (ref: 05:00–08:59 am)					
09:00–12:59 am	0.030	−0.005	0.065	0.087	0.03
01:00–04:59 pm	0.074	0.038	0.110	<0.001	0.08
05:00–09:00 pm	0.086	0.043	0.130	<0.001	0.09
Days in milk (DIM)	<0.001	<0.001	0.001	<0.001	0.14
Separation of diseased/lame cows (ref: never)					
Sometimes	0.103	0.052	0.152	<0.001	0.65
Always	0.176	0.117	0.234	<0.001	0.76
Housing type (ref: raised cubicles)					
Deep bedded cubicles	−0.049	−0.106	0.009	0.096	0.30
Straw yard ^1^	−0.084	−0.136	−0.031	0.003	0.53
Active habituation of heifers to milking/AMS	0.085	0.040	0.129	0.001	0.61
Cow:cubicle ratio or lying area ^2^ (m^2^/cow) (ref. suboptimal)					
According to recommendations	0.060	0.009	0.112	0.025	0.43
Generous	−0.132	−0.209	−0.055	0.002	0.58
(Also) manual concentrates provision	0.054	0.003	0.106	0.042	0.39
Contact time per cow ^3^ (min/d)	−0.006	−0.010	−0.002	0.004	0.54
VIF = 1.018–2.453					

^1^ six farms with straw yards and three farms with a combination of straw yards and deep bedded cubicles. ^2^ based on recommendations cited in the literature (details in Appendix Table A2 and Table A3). ^3^ including milking, excluding time near the cows, but on machines.

**Table 4:** Descriptive data of cows’ behaviors towards humans, milk yield, and udder health at animal level; and univariable associations with the cows’ FCM levels (ng/g), analyzed by means of Spearman rank correlations (r_s_).

Variables	Descriptive Data				Associations with the Cows’ FCM Levels	
Cow Behavior ^1^	*n*	Mean ± sd	Median	Min–Max	r_s_	*p*
AD (cm)	569	26.8 ± 35.1	10.0	0.0–170.0	−0.069	0.102
QBAanimal (PC1) **^2^**	315	−0.090 ± 1.025	−0.178	–1.713–2.355	−0.149	0.008
**Milk Recording Data ^3^**	***n***	**Mean ± sd**	**Median**	**Min–Max**	**r_s_**	***p***
ECM (kg)	674	25.22 ± 6.70	25.11	7.93–43.60	−0.034	0.385
SCS	674	3.08 ± 1.70	2.90	−0.64–9.25	0.109	0.005

^1^ associations with values of the first FCM measurement (when cow behavior was also recorded). ^2^ negative QBA values relate to relaxation/attraction/trust, positive values relate to fear/distress/ aversion. ^3^ associations with medians of repeated FCM measurements.

## Appendix A

**Table A1:** Spearman rank correlations (r_s_) between metric farm factors considered in multivariable analysis.

Variables		Max. No of Cows per Group	Concentrates (kg/Cow*Year)	No of Cows per Stockperson	Contact Time per Cow	Voluntary Contact to Cows (%)	Voluntary Contact to Dry Cows (%)
Herd size	r_s_	0.895	0.106	0.458	−0.249	−0.146	−0.238
	*p*	0.000	0.615	0.021	0.229	0.487	0.253
Max. no of cows per group	r_s_		0.104	0.445	−0.360	−0.209	−0.274
	*p*		0.620	0.026	0.077	0.316	0.185
Concentrates (kg/cow*year)	r_s_			0.315	−0.008	−0.047	−0.014
	*p*			0.125	0.968	0.824	0.946
No of cows per stockperson	r_s_				−0.544	−0.252	−0.357
	*p*				0.005	0.225	0.080
Contact time per cow	r_s_					0.389	0.456
	*p*					0.055	0.022
Voluntary contact to cows (%)	r_s_						0.884
	*p*						0.000

**Table A2:** Lying space—recommendations for herds with a milking parlor or automatic milking system (AMS) and with horned or hornless cows.

References	Cubicles (Milking Parlor) (Cow:Cubicle Ratio)	Cubicles (AMS) (Cow:Cubicle Ratio)	Straw Yards (Hornless Cows) (m^2^/Cow)	Straw Yards (Horned Cows) (m^2^/Cow)
[59]	1:1		4.5–5 m^2^	7–9 m^2^
[60]	1:1		6 m^2^	8 m^2^
[61]	1:1	1:0.98^1^	8–9 m^2^	
[62]			≥5 m^2^	8 m^2^
[63]				8 m^2^
[64]		1:0.90^2^		

^1^ calculated according to [61]. ^2^ calculated according to [64].

**Table A3:** Lying space—categories for analyses derived from recommendations.

Categories	Cubicles (Milking Parlor) (Cow:Cubicle Ratio)	Cubicles (AMS) (Cow:Cubicle Ratio)	Straw Yards (Hornless Cows) (m^2^/Cow)	Straw Yards (Horned Cows) (m^2^/Cow)
Suboptimal	<1:1	<1:0.95	<4.5 m^2^	<7 m^2^
According to recommendations	1:1–1:1.05	1:0.95–1:1	4.5–6.5 m^2^	7–9 m^2^
Generous	>1:1.05	>1:1	>6.5 m^2^	>9 m^2^

**Table A4:** Feeding places—recommendations for herds with a milking parlor or automatic milking system (AMS) and with horned or hornless cows.

References	Feeding Gates (Milking Parlor) (Cow:Feeding Place Ratio)	Feeding Gates (AMS) (Cow:Feeding Place Ratio)	Feed Rails (Hornless) (Feeding Place Width)	Feed Rails (Horned) (Feeding Place Width)
[59]	1:1		70–75 cm	
[60,65]			75 cm	85–90 cm
[61]	1:1	1.5:1	65–75 cm	
[62]			75 cm	85 cm
[63]	1:1–1:1.1			85–100 cm
[64]		1.5:1		
[66]	1:1.1			85 cm

**Table A5:** Feeding places—categories for analyses derived from recommendations.

Categories	Feeding Gates (Milking Parlor) (Cow:Feeding Place Ratio)	Feeding Gates (AMS) (Cow:Feeding Place Ratio)	Feed Rails (Hornless, Milking Parlor) (Feeding Place Width)	Feed Rails (Hornless, AMS) (Feeding Place Width)	Feed Rails (Horned, Milking Parlor) (Feeding Place Width)	Feed Rails (Horned, AMS) (Feeding Place Width)
Suboptimal	<1:0.95	<1:0.83	<70 cm	<62 cm	<80 cm	<70 cm
According to recommendations	1:0.95–1:1.05	1:0.83–1:1	70–75 cm	62–75 cm	80–90 cm	70–90 cm
Generous	>1:1.05	>1:1	>75 cm	>75 cm	>90 cm	>90 cm
